# Lip Reading by Alternating between Spatiotemporal and Spatial Convolutions

**DOI:** 10.3390/jimaging7050091

**Published:** 2021-05-20

**Authors:** Dimitrios Tsourounis, Dimitris Kastaniotis, Spiros Fotopoulos

**Affiliations:** Department of Physics, University of Patras, 26504 Rio Patra, Greece; dkastaniotis@upatras.gr (D.K.); fotopoul@upatras.gr (S.F.)

**Keywords:** lip reading, temporal convolutional networks, spatiotemporal processing

## Abstract

Lip reading (LR) is the task of predicting the speech utilizing only the visual information of the speaker. In this work, for the first time, the benefits of alternating between spatiotemporal and spatial convolutions for learning effective features from the LR sequences are studied. In this context, a new learnable module named ALSOS (Alternating Spatiotemporal and Spatial Convolutions) is introduced in the proposed LR system. The ALSOS module consists of spatiotemporal (3D) and spatial (2D) convolutions along with two conversion components (3D-to-2D and 2D-to-3D) providing a sequence-to-sequence-mapping. The designed LR system utilizes the ALSOS module in-between ResNet blocks, as well as Temporal Convolutional Networks (TCNs) in the backend for classification. The whole framework is composed by feedforward convolutional along with residual layers and can be trained end-to-end directly from the image sequences in the word-level LR problem. The ALSOS module can capture spatiotemporal dynamics and can be advantageous in the task of LR when combined with the ResNet topology. Experiments with different combinations of ALSOS with ResNet are performed on a dataset in Greek language simulating a medical support application scenario and on the popular large-scale LRW-500 dataset of English words. Results indicate that the proposed ALSOS module can improve the performance of a LR system. Overall, the insertion of ALSOS module into the ResNet architecture obtained higher classification accuracy since it incorporates the contribution of the temporal information captured at different spatial scales of the framework.

## 1. Introduction

Convolutional Neural Networks (CNNs) have been used successfully in single image recognition tasks, like classification [1], detection [2], and segmentation [3], while lately they have been started to become a promising selection for visual sequence modeling tasks too [4,5,6]. Therefore, CNNs have been recently evaluated in sequence modeling tasks with various data types including amongst others audio synthesis [7], language modeling [8], machine translation [9] as well as image sequence modeling [10]. In this manner, the extraction of image-based features happens independently from each frame of the sequence using a 2D CNN, and then, a sequence feature-level modelling stage is utilized, such as either the simple pooling of the features or the implementation of a recurrent architecture, to predict the video action. These image sequence modeling tasks are related to video understanding, like activity recognition [11] or Lip reading [12,13,14]. A natural extension of the spatial (2-Dimensional) convolutions for video is the spatiotemporal (3-Dimensional) convolutions in order to incorporate both spatial relations of each image frame and temporal relations between continuous frames [10]. Despite the progress in the representation learning from image sequences, significant progress has been also made by adopting feedforward architectures for the task of sequence encoding. Architectures based on Temporal Convolutional Networks (TCNs) tend to replace the recurrent architectures since TCNs exhibit several interesting properties as they can hold long-effective memory, introduce more layers as well as explicit depth, and learn in a feedforward manner with stable gradients [15]. Additionally, TCNs offer significant improvements implementation-wise, as they can parallelize better the processing load [4]. Thus, the TCNs gain ground over conventional approaches for visual sequence modeling, such as the Long Short-Term Memory networks (LSTMs) and Gated Recurrent Units (GRUs), mainly due to their applicability advantages [4].

In the problem of automatic Lip Reading (LR), also known as Visual Speech Recognition (VSR), the objective is to recognize a spoken word using visual-only information from the speaker. This information is mainly focused on the mouth region [12] which is the optical output of the human voice system. However, several approaches investigate the contribution of other parts of the face, as for example the upper face and the cheeks [16]. The LR problem can be formulated as an image sequence modeling task in which frames provide information about different phonemes that in a particular sequence compose a world.

**Figure 1:**
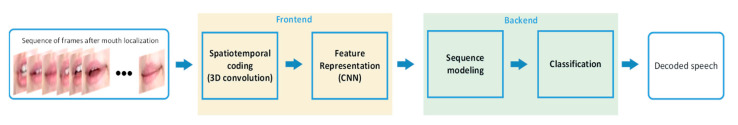
The workflow in the recent Lip Reading (LR) systems based on deep learning. Given an image sequence, the mouth regions are detected and cropped. Then, the frontend consists of a spatiotemporal convolution stage along with a CNN for capturing the short-term dynamics and finally extracting a feature vector for each frame. The features are the inputs of the backend that is composed of the sequence modeling and classification stages. Thus, the information is encoded by a sequence modeling stage, and the output of the sequence modeling is being fed to the classification module, which provides the class probabilities, i.e., the decoded speech.

A typical LR system based on deep CNNs is composed by a processing pipeline utilizing two basic components, the frontend and backend, as illustrated in the Figure 1. The frontend is dealing with the feature mapping in a vectorial representation of the input image sequence while the backend refers to the sequence modeling [17]. Thus, firstly, the frames from a spoken speech time interval are processed in order to detect and isolate the region of interest, i.e., the mouth area. In some cases, the face sequence is aligned beforehand ensuring that the mouth area is seen normalized by the network. Next, the mouth image sequence is fed to a spatiotemporal module with 3-Dimensional (3D) convolutions to extract both spatial and temporal information. Then, a CNN is utilized as a feature extractor for each frame independently. The obtained feature vectors are the inputs of a sequence-to-sequence mapping with a sequence modeling encoding method, such as Recurrent architectures [16], [18,19,20] or Temporal Convolutional Networks (TCNs) [21,22] in recent time. The output of the sequence modeling usually caters a fully connected network to provide the class probabilities and thus the predicted spoken speech. Thus, the sequential data is encoded via the spatiotemporal stage to extract low level features that are learned in the first layers while followed by the frame-wise vectorial representation at the frontend and the sequence modeling stage at the backend for vectorial sequence mapping [23].

In our work, we propose a novel LR system focusing on the design of the spatiotemporal module in the entrance of the framework. Our motivation stems from the fact that the spatiotemporal information in the image sequence is captured using temporal pooling layers and 3D convolutional filters. In this context, we designed a module that consists of both 3D and 2D convolutional layers, instead of pooling layers, in order to summarize the temporal as well as the spatial dependency of the input image sequence and make amends for the limitations of using only 2D operations. The proposed LR system introduces a convolutional-based spatiotemporal module for capturing the short-term dynamics between image frames in the beginning, along with parallel identical CNNs that applied in frame-level subsequently, and finally followed by a TCN that encodes the frame-related feature vectors—extracted by the CNNs—in order to provide the spoken word in the end. Our approach exhibits several interesting properties. Thus, our contributions are summarized as follows: (1) we encode both spatial and spatiotemporal information directly on image space using a novel module that utilizes the benefits of two words by alternating 2D and 3D convolutions, and its incorporation into a LR system can improve the performance; (2) we show that capturing the spatiotemporal information at different spatial scales of the framework is important for the efficiency of a LR system; and (3) we make use of a TCN applied in vectorial representation of image sequence for sequence modelling enhancing this last effective breakthrough in LR systems. By following our novel scheme for designing a LR system, the dynamics of the lip motion are better captured during the feature extraction process as the system is able to learn relations between neighboring images of the sequences in the input layer (using ALSOS) and then relations between sequential feature maps (using TCN). The proposed LR system is tested on a dataset designed to approach a clinical scenario, where a person has lost the ability to speech and can only communicate with gestures as well as lip’s movements, utilizing Greek words [21] and further verified and compared on a standard large-scale benchmark of English words, namely, the LRW dataset [24]. The importance of the proposed spatiotemporal module lies on taking advantage of a deep topology that can capture short-temporal characteristics of input sequence, and the efficiency of the module is showed since its incorporation in the framework improves the performance of the LR system in two different languages, Greek and English.

## 2. Related Work

In the early days of LR, research works were focused on relatively simple recognition tasks such as the digit or alphabet classification where the number of classes is small, due to the limited efficiency of the algorithms [25,26,27]. Nowadays, LR is extended in the more complex tasks of word and sentence recognition and driven by the capacity of deep learning models, LR is focusing on real-world problems. Towards this direction, larger datasets are nowadays utilized even in unconstrained conditions [24,28]. The LR in terms of word level recognition is the task of estimating the spoken word from one input image sequence. The reason behind the wide use of word-level recognition in the LR research community is the challenging nature of the task but also that it can be studied under secured environment. This makes the task suitable for research investigation (by designing a dataset and studying the ability of automatic LR systems), whereas the phrase or sentence recognition is a concern for a more practical but a more difficult to study scenario. The design of LR systems has a long history; whilst the early works are based on the hand-crafted features, the deep neural networks provide a noteworthy improvement in the performance [12]. In the pioneer work of Chung and Zisserman [24], deep CNNs were used in the problem of LR for the first time, and among others, this is due to the large-scale word-level LR database that is introduced in the same work. The effectiveness combination of ResNet [29] with Recurrent architectures for the task of LR was later demonstrated by Stafylakis et al. [18]. Both works [18,24] proposed the use of spatiotemporal convolutions, i.e., 3D-convolutions, as a fundamental learning module in the beginning of the LR system. In this manner, the deployment of CNNs stemming from the ResNet family combined with spatiotemporal convolutions in the frontend and Recurrent architectures in the backend established a breakthrough in an efficient LR system. This led to many variational implementations such as the single-stream Residual network or the two-stream ResNet, which fed with images and optical flow, along with a bi-directional Long Short-Term Memory network (BiLSTM) [20,30]. The combination of 3D-convolution with ResNet and Bi-directional Gated Recurrent Units (BiGRU) exploiting the gradient policy [31], mutual information [32], deformation flow [33], and various image augmentation techniques [34] is evaluated on the word-level LR task achieving state-of-the-art results. Moreover, the work of Zhang et al. [16] pointed out the importance of face alignment and extraoral facial regions beyond the lips in the overall efficiency of the visual speech recognition. A strong differentiation point in the LR approaches is the utilization of Temporal Convolutional Networks (TCNs) in order to replace the Recurrent architectures (LSTM and GRU) for sequence modelling. Variations of TCNs are being recently evaluated at the backend of LR systems [21,22], providing better performance, more stability, and much lower computational cost than the Recurrent blocks. Therefore, TCNs seem to be the new fundamental component in the design of a LR system.

Despite the success of 2D CNNs operating on spatial domain, the applicability of 3D CNNs, working on spatiotemporal domain, in sequence learning tasks is more efficient [35]. The spatiotemporal convolutions can capture the short-term dynamics and can be beneficial in Lip reading even when a sequence modeling network is utilized too [20]. Shallow 3D CNNs have shown significant improvements in video-based tasks, like action recognition or Lip reading [17,36]. Although a deeper 3D CNN is expected to do better than a shallow one, the deep 3D CNNs require very large datasets to generalize well, like the Kinetics, or inflate the pre-trained weights of an efficient 2D CNN into the 3 dimensions for initialization [20,36,37]. The most LR systems incorporate 3D convolutions, but only up to three 3D convolutional layers are utilized due to the easily overfitting of deeper topologies, when the number of parameters is increased. As far as the authors’ knowledge, only the recent work of Weng et al. [20] used a deep spatiotemporal stage, beyond the three layers, in a LR system. It is worth to notice that in all the aforementioned works, the spatiotemporal convolutions are applied only in the input image sequence and not in the later stages of the feature representations learnt by the CNN. Thus, there is room for further investigation in this direction. Our main contribution is a newly proposed learnable module (called ALSOS—alternating spatiotemporal and spatial convolutions) that combines spatial and temporal information by synthesizing 3D and 2D convolutional layers into a deep spatiotemporal module. This module is fed with the input image sequence as well as it is applied in intermediate positions of the frontend of the LR system. In our work, different implementations of the proposed spatiotemporal module in conjunction with ResNets are utilized in the frontend along with TCNs and fully connected convolutional layer in the backend of the LR system. Finally, our experimental results in the word-level LR task show that the incorporation of the proposed deep spatiotemporal module delivers a performance boost of the system. Moreover, the experiments reveal that the more effective utilization of ALSOS is observed when the spatiotemporal convolutions are added in between ResNet-18 blocks as compared with approaches that just stack multiple spatiotemporal convolutions. This is an upside of our method since it allows to incorporate the temporal information at all spatial scales of the framework, something that the existing methods have not already explored in the LR task.

## 3. Proposed Method

Our proposed module is named ALSOS (alternating spatiotemporal and spatial convolutions) and relies on alternating spatiotemporal as well as spatial convolutions to efficiently encode the input image sequences. Initially, the input sequence is passed through the ALSOS, and then, the extracted feature maps are processed either by another ALSOS module or a ResNet CNN. Three main topologies based on ALSOS and ResNet, namely ALSOS-ResNet, are utilized in the frontend. The backend consists of a TCN that encodes high-level sequence information, while the final classification is provided by a fully connected layer and a Softmax activation. The grounds for building the ALSOS module are that temporal information needs to be taken into consideration at different levels of feature representation and not only at the beginning, i.e., in the input of the image sequence where the feature learnt correspond to low-level visual features or the end (i.e., in the sequence modelling of the backend) of the framework where spatial information is eliminated. Additionally, we assume that depth information can potentially be delivered by spatial only information, and the temporal information can be exploited in different layers of a Deep CNN. In this manner, the temporal information encoded in the features both in frontend and backend using the spatiotemporal module as well as the TCN, respectively. Thus, the overall architecture is built solely with feedforward convolutional layers, and it can be trained in an end-to-end fashion.

### 3.1. Alternating Spatiotemporal and Spatial Convolutions (ALSOS) Module

**Figure 2:**
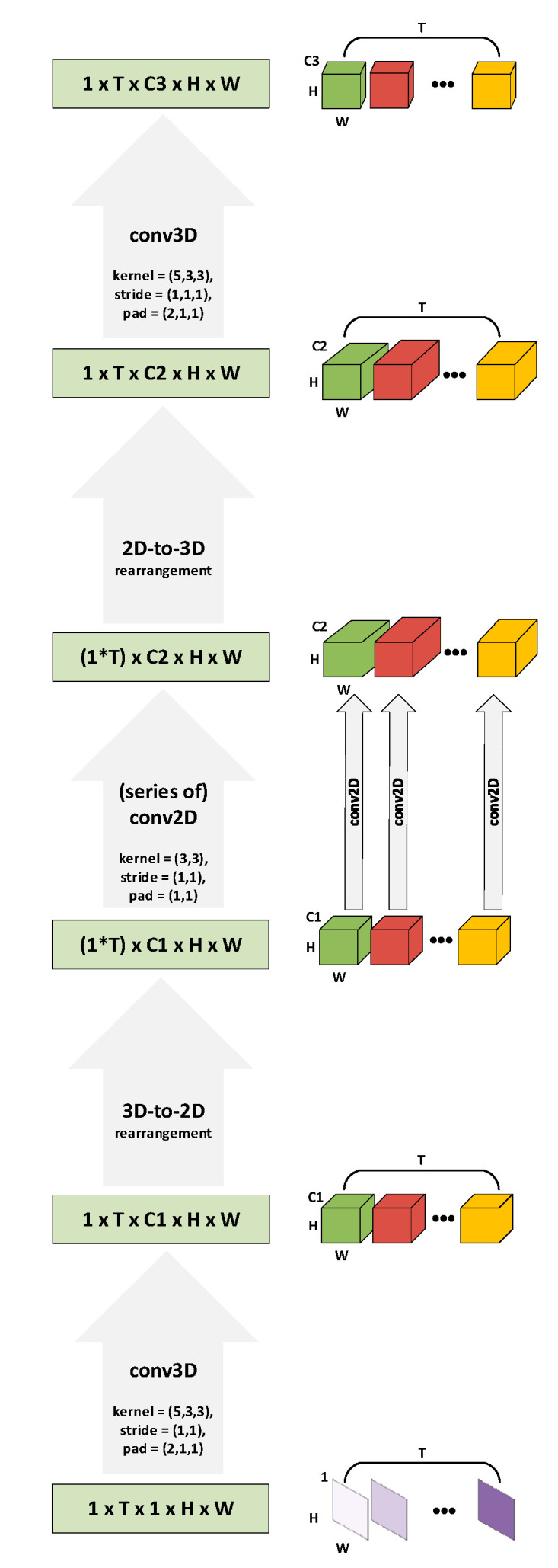
The ALSOS module. The flowchart of one input sequence is demonstrated from bottom to top while the procedure steps (gray arrows) as well as the size of the data volumes (green boxes) are represented on the left side, and the corresponding volumes are presented on the right side of the scheme. The H and W correspond to spatial sizes (height and width) while C1, C2, and C3 are the depth sizes (number of channels) of the volumes and the T is the length of the sequence (with the symbol (*) represents the scalar multiplication operation). Ultimately, the ALSOS module provides a sequence-to-sequence mapping utilizing both spatiotemporal and spatial convolutions.

ALSOS is a sequence-to-sequence mapping module, in a manner that it can map any sequence to a new one preserving the sequence length but transforming the size (i.e., Height, Width, and Channels) of the sequence elements. Therefore, the ALSOS module composed of 3D convolutions (spatiotemporal), 2D convolutions (spatial), and two conversion components, i.e., 3D-to-2D and 2D-to-3D conversions that enable the translation of a sequence to groups of feature maps and conversely. The data flow inside the ALSOS module is presented in Figure 2. One input image sequence is noticed as $X_{j}=\left\{x_{1},\ldots ,x_{T}\right\}$, where $x_{i}\in {\mathbb{R}}^{CxHxW}$ is an image (frame) of the sequence with the W, H, and C correspond to the width, height, and depth (number of channels) of the volume (i.e., image-frame), respectively, while i={1,..,T} with $T\in {\mathbb{R}}^{+}$ is the length of the sequence, and j corresponds to the selected sample (sequence). Considering that the color information of images is not crucial for the LR task [22,24], grayscale images are utilized at our implementation, and thus, the depth of each frame equals to one C=1. Moreover, when a batch of sequences is available, the input volume is noted as $G=\left\{X_{1},\ldots ,X_{z}\right\}$ for $X_{j}$ representing one image sequence and j={1,..,Z} with $Z\in {\mathbb{R}}^{+}$ corresponding to the length of the batch. The exploitation of batch of samples (i.e., set of image sequences) is necessary for the efficient training with the backpropagation algorithm. Therefore, for a batch of Z input sequences, the corresponding volume has [Z x T x C x H x W] dimensions. Given the fact that grayscale images are used, the C is 1 (C=1), and in the case of one input sequence, the Z equals to 1 (Z=1), then the input degenerates to [1 x T x 1 x H x W] dimensions.

At the first step, the input sequence is filtered using a 3D kernel with spatial size 3 × 3 pixels and temporal size equal to 5 in order to learn the relations between each 5 sequential frames. The temporal interval is set to 5 neighboring images after experimental investigation of different values and under the case of encoding short-term dynamics of input sequence.

The output of the 3D convolution step is rearranged with a 3D-to-2D conversion because our purpose is to handle each temporal instance as a segregated group of feature maps. In this manner, the 3D-to-2D rearrangement disconnects the feature maps from each temporal instance that corresponds to each image-frame. To do this technically, the T frames (T temporal instances) are considered as a batch of T volumes of feature maps, which can be noted as $B=\left\{V_{1},\ldots ,V_{T}\right\}$, where $V_{q}\in {\mathbb{R}}^{C1xHxW}$ is the feature maps that rely on the q frame with q={1,..,T} and C1 being the number of used kernels from the previous step.

The feature maps that depend on every frame (temporal instance) are processed independently by 2D convolution kernels. Thus, the 2D convolutions are applied in a frame-wise concept. In such way, a series of 2D convolutions is executed since in each temporal instance (in each group of feature maps) is implemented a number of C2 filters (C2 is the number of selected 2D kernels of the current step). More specifically, the 2D convolution procedures are calculated in each volume $V_{q}$ that corresponds to each frame of the original sequence.

A 2D-to-3D rearrangement is implemented next, taking into account the length T of the sequence. The 2D-to-3D conversion reconnect the produced feature maps based on the temporal order of the original sequence to provide a new sequence. Thus, the output of this 2D-to-3D step is a new sequence $Y_{j}=\left\{y_{1},\ldots ,y_{T}\right\}$, where $y_{i}\in {\mathbb{R}}^{C2xHxW},\;i=\left\{1,..,T\right\}$, and C2 are the depth of each element of the sequence that is determined by the previous step of 2D convolutions.

The last step of ALSOS module is a spatiotemporal (3D) convolution procedure that learns the final sequence mapping and provides the final sequence $Z_{j}=\left\{z_{1},\ldots ,z_{T}\right\}$, where $z_{i}\in {\mathbb{R}}^{C3xHxW},\;i=\left\{1,..,T\right\}$, and C3 is the output depth of sequence’s elements.

As we can notice from Figure 2, the spatial dimensions of images (H, W) are selected to not modified during the processes of ALSOS (by applying the proper image padding at the steps that it needs), while the depth of the feature maps (the number of kernels in each step, i.e., C1, C2, C3) is varied. These three parameters along with the dimensions of kernels (spatial and/or temporal) are defined in each case based on the best performance of the overall system, but as we observed from our experiments, they did not have major influence. For completeness, the utilization of ALSOS can be yet extended using a batch of sequences as input with the same logic, and in that case, the batch size Z is different from 1 (i.e., Z>1).

The proposed ALSOS is an independent and portable module that can be used as addendum inside a topology in many ways. Thus, it can be utilized for composing deeper architectures by stacking multiple ALSOS modules one after the other in a sequential way or by placing ALSOS modules between different 2D convolutional layers allowing network to incorporate spatiotemporal information. In this way, by switching back and forth to sequence and independent feature maps, the 3D and 2D convolutions can be combined, and the topology can be trained end-to-end.

### 3.2. ALSOS and CNNs: Architecture for Image Sequence Mapping

Our objective is to utilize the ALSOS module for the task of Lip reading. The typical implementation includes the ALSOS in the position of the spatiotemporal coding stage. In this context, the ALSOS module deals directly with the input images and provides the mapping of the input image sequences into sequences of higher-level representation vectors incorporating the short-term information between short-time consecutive frames. Hence, the following 2D convolutional layers can capture implicitly spatiotemporal dynamics too. In this case, the spatiotemporal stage consists of the sequential ALSOS modules—one module after the other—and at the final ALSOS a 3D-to-2D rearrangement is placed at its top in order the output can be processed from a standard CNN architecture, e.g., the ResNet, in each temporal instance (i.e., frame-by-frame). Next, the backend of the LR system is responsible for the sequence modeling and the prediction of the spoken word.

In a variation of this scheme, the CNN can be removed and replaced with additional sequential ALSOS modules in a manner that the spatiotemporal convolutions can compensate for the CNN. In this second case, the feature extraction is simply implemented via the last ALSOS, which is modifies properly by attaching a proper setting of 3D-to-2D rearrangement in order to extract the required sequence of feature vectors.

In a slightly different approach, the ALSOS module can used in between 2D convolutional layers, e.g., intermediate ResNet layers’ blocks. Given that the ResNet blocks are fed with images (and not with image sequences), a 3D-to-2D rearrangement is necessary after each ALSOS module. Under this third configuration, the CNN takes as input the result of ALSOS (after the added 3D-to-2D step) and operates in a frame-by-frame manner. In this context, the CNN is producing a vector representation corresponding to every frame of the sequence, encoding in this manner not only frame-level information but also involving implicitly information about relations between different frames due to previous spatiotemporal operations. This variant of the ALSOS seems to be the most effective for the task of LR (based on the experimental results that demonstrated on the next section). This is however expected as it combines the strengths of ResNet in learning efficient feature representations with the temporal information learnt by the 3D convolutions of ALSOS.

**Figure 3:**
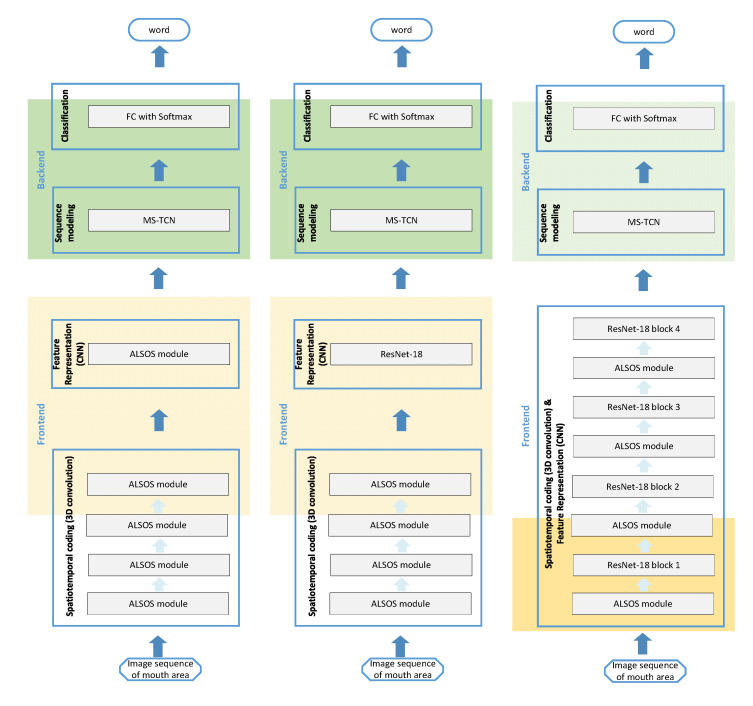
The three proposed LR systems based on ALSOS and ResNet variants. The ALSOS module can be utilized as the initial and/or the intermediate building block of a convolutional-based topology at the frontend, allowing to design three combinations of ALSOS and CNN that result to three different LR systems. In all LR systems, the backend consisted of a TCN and a Fully Connected layer (FC) using Softmax for the word prediction. (**Left**) topology: an architecture based solely on ALSOS modules at the frontend. (**Middle**) topology: stacked ALSOS modules as spatiotemporal coding stage followed by a ResNet at the frontend. (**Right**) topology: modified ResNet to include ALSOS modules in between ResNet blocks at the frontend.

These three ALSOS based LR systems are presented in the Figure 3, and they constitute the three core directions to incorporate a spatiotemporal module in the frontend of the framework. The first described combination is illustrated in the middle of Figure 3 (typical case where ALSOS works as the spatiotemporal coding stage) while to the left is the second referred combination (where the frontend is utilized just with ALSOS modules) and the third proposed architecture is presented to the right (where ALSOS is included between ResNet blocks). It is good to mention here that the number of stacked ALSOS modules in each system is defined after due diligence in order to provide the best performance. The backend of the three designed LR systems is fed with the vectorial representations of input sequence and provides the sequence modeling process. In this manner, the vectors produced for each frame need to be mapped to a final feature vector representing the sequence and next, this final feature vector needs to provide a mapping to one word. To achieve this, a non-causal TCN is used for learning the sequence modeling from sequence-to-sequence [21,23]. The TCN’s outputs are handled with an attention mechanism through averaging in order to extract the final feature vector. This final vector is mapped to one of the words via fully connected layer with Softmax activation that acts as a classifier. All the different LR systems based on the ALSOS and CNNs variants are utilized with the cross-entropy loss that is applied to the output of the fully connected layer in the backend, and thus, the whole system can be trained using an end-to-end learning scheme.

The main advantages of the proposed LR system are: (a) the spatial information is naturally combined with the temporal information using the consecutive 3D and 2D convolutions of ALSOS; (b) the learning process is more stable since solely convolutional layers are utilized as opposed to recurrent architectures that can suffer from exploding or vanishing gradients; (c) the amount of weights scales well with the length of the input sequence, (d) for the many-to-one mapping and in particular for the image sequence classification (where the input sequence is asked to be mapped into a single class), the size of memory required is minimized; and (e) the dilated convolution in combination with the temporal down-sampling over time of TCN allows the model to capture long effective history.

The exploitation of an ALSOS-based approach is expected to be beneficial for the LR task since LR is purely based on the spatiotemporal patterns, such as the combination of lips, teeth, tongue, and mouth opening, and the relation between neighboring frames. The ALSOS module utilizes the benefits of alternating between spatiotemporal and spatial convolutions, combining the best of two worlds. Thus, the temporal dynamics encoded in the features that extracted from the ALSOS are impaired by the following 2D CNN for frame-wise feature representation and in conjunction with the frame-by-frame sequence modelling; the whole framework can capture the spatiotemporal characteristics of visual LR.

## 4. Experimental Results

### 4.1. Materials and Methods

The proposed LR architecture consists of the ALSOS module and the CNN model of ResNet-18 in the frontend as well as the Multi-Scale Temporal Convolutional Network (MS-TCN) and one fully connected convolutional layer (FC) in the backend. The weights of the ALSOS (3D and 2D convolutions) are always initialized with the He Normal initialization using a uniform distribution [38]. When ALSOS is used in-between ResNet blocks (in between changes in the number of channels [29]), the number of channels across all convolutions within the ALSOS module is equal to the number of channels extracted from the previous ResNet layer. The ALSOS module has temporal kernel size equal to 5 and spatial kernel size equal to 3 with padding equal to 2 and 1, respectively, for 3D and 2D convolutions. The stride is always equal to 1 in all ALSOS modules. The ResNet model (2D convolutions) is pretrained on the ImageNet, and it is available from Pytorch model zoo [39]. These 2D convolutions are implemented here using a kernel size equal to 3 and padding equal to 1. The weights of the MS-TCN (1D convolutions) and the fully connected convolutional layers (1D convolutions) are initialized with He Normal initialization [38]. Since training from scratch is not attainable [20], the whole system can pretrain end-to-end either using a random subset of 20 words from LRW-500 [21] or increasing progressively the training samples up to the total classes of LRW-500 dataset as warm up [17], before the final training in the target dataset if any. All experiments performed using the PyTorch framework [39].

The importance of hyperparameters is well known in machine learning and especially in the Deep learning era. However, it is almost impractical to perform hyperparameter search due to limitations imposed by the amount of computational resources required. The learning rate and weight decay was set following the work of Martinez et al. [22], which utilize a similar architecture with just one 3D kernel (5 × 7 × 7) before the ResNet-18 model and a MS-TCN followed by one FC with Softmax. Thus, the whole system is trained using the AdamW optimizer [40] with the learning rate set to 0.0003, the weight decay to 0.0001, and with the maximum possible mini-batch for 50 epochs. The training was performed on four GeForce RTX 2080 GPUs, each one with 8GB memory, and the total training time for 50 epochs was approximately 50 h.

The computational complexity and the processing time for the three proposed LR systems (Figure 3) and the LR system of Martinez et al. [22] are demonstrated in Table 1. The LR system built with ALSOS modules (without the ResNet) has the biggest number of operations (and proportionally processing time), since it implemented the larger number of ALSOS modules. The LR from [22] exhibits the smallest processing time, something expected since it calculates only one 3D convolution. The replacement of this one 3D convolution with four sequential ALSOS modules in order to design our proposed ALSOS & ResNet-18 + MS-TCN system increases the processing time by about 4.6ms (about 30%), whilst improving the performance as we discuss in the next sections. Finally, the alternating of ALSOS modules and ResNet-18 blocks slightly increased the number of parameters whereas the processing time was reduced by 2.3 ms (about 15%), and probably, this is due to the presence of residual connection into ResNet blocks.

### 4.2. Datasets

For our evaluation purposes, two Lip reading datasets in Greek [21] and English [24] languages are used. Both datasets are generated for word-level Lip reading. The first dataset adopts a biomedical and clinical case since the selected words are useful for a patient during medical treatment and the environmental conditions during recordings are aligned with this scenario. The second dataset is among the largest and most challenging LR databases since its samples result from an automatically cropping procedure of video clips recorded from BBC TV broadcasts.

The Greek dataset LRGW-10 [21] is a relatively small database but very challenging since the environmental conditions are varied, and the length of the sequences is not fixed. The speakers are 10 (6 males and 4 females), and pronounce 10 words, not less than 3 times and up to 10 times per word. Εach test and validation set includes one sequence per word, and the rest of sequences are used for the training set. The difficulty of this dataset is that during the generation of videos, the participants used front camera of their smartphone, and thus, all manner of recording conditions can occur, such as the distance from the camera, the lens specifications, the lighting conditions, etc. Moreover, another challenging point of the LRGW-10 dataset is the variable length nature of the data since every word has a different number of frames, and even the same word has a large dispersion of length between various repetitions or speakers. The mouth region is detected by using a mouth detector and then cropped with an extra 10% of the width and height, as proposed by the generators of the dataset [21]. In our experiments, the cropped mouth images are resized to 112 × 112 pixels and converted to grayscale images.

The English dataset, namely LRW-500 [24], is a challenging large-scale database containing 500 different words with a sum of 488,766 training samples, 25,000 samples for validation and 25,000 samples for test. Every word is represented from a video of 29 frames, in a manner that the word is always in the center of the video clip. Since, the data are originated from TV programs, the words are not fully isolated and maybe the first and the last frames can contain information from other words. In our evaluation, the face images of LRW were aligned by using the mean face alignment procedure proposed by Martinez et al. [22]. Finally, the images were cropped to 88 × 88 pixels resolution and converted to grayscale images.

### 4.3. Evaluation on Greek Words in a Biomedical Application Using the LRGW-10 Dataset

In this section, we are investigating the efficiency of the proposed system in a real-word problem related to clinical conditions using the LRGW-10 dataset [21]. Towards this goal, the three variants of ALSOS and ResNet-18 presented in Figure 3 are evaluated. To study the effectiveness of each component in the overall performance, the weights of the learnable components are initialized and pretrained under various conditions. Since it is known that the training of a LR system from scratch is not easy with a small number of training samples and can lead to suboptimal results [22], we pretrained our LR system both with a randomly selected subset of 20 words from LRW dataset and with the total training set of LRW-500. In this pretraining phase, the model weights (of ALSOS, TCN, and Fully Connected layer) were initialized following He Normalization, except the ResNet which is always initially pretrained on ImageNet. Next, the second-round training is performed on the LRGW-10, and the test classification accuracy results are presented in Table 2. Moreover, Table 2 contains the comparison with the results from the work of Kastaniotis et al. [21] in this dataset.

Several interesting conclusions can be drawn observing the results of Table 1. The insertion of ALSOS module in between the layers (blocks) of ResNet Figure 3—Right) provides superior performance compared to the architecture that uses the stacked ALSOS modules (Figure 3—Left) and the topology of stacked ALSOS modules followed by the ResNet (Figure 3—Middle). The best results obtained from mixing ALSOS modules and ResNet layers’ blocks probably have to do with the presence of residual connections that enable more efficient learning of ALSOS’ weights (providing larger gradients) and avoiding overfitting. For the later LR system, the different pretraining conditions are evaluated. Finally, the pretraining of the system with the auxiliary but relative LRW-500 dataset is beneficial, and the accuracy is increased over 4% up to the highest value of 56.3%. It is also worth noticing that pretraining with only a small subset of LRW-500 by randomly selecting the sequences from 20 words seems adequate, considering that the corresponding LR system achieved just 2% lower accuracy (54.3%), as compared to the case of training on all 500 words of LRW (56.3%), while using only 4% of LRW training samples.

### 4.4. Evaluation on English Words in the Large Scale LRW-500 Dataset

The mini-batch size is a crucial hyperparameter for the training efficiency of a deep learning architecture, and empirically, the larger the minibatch size provides better results since the smaller the mini-batch, the less accurate the estimation of the gradient will be. In the next Table 3, the mini-batch size was estimated by measuring the performance on the validation set of LRW-500 dataset for the following mini-batch sizes: 8, 16, 32, and 40 (which was the maximum possible value for our GPUs environment). It is evident that the mini-batch size can significantly affect the performance of the LR system; however, it cannot be fully investigated as going beyond batch size of 40 sequences was not feasible. Therefore, thew training on LRW-500 dataset is implemented with mini-batch size of 40 sequences for the next experiments.

Now, we present an overview of the LR systems that evaluated the challenging LRW-500 dataset in a way that the reader can have a general outlook of the field as well as our implementation on this popular dataset. The LRW-500 dataset was created by Chung and Zisserman [24] in 2016, allowing researchers to evaluate their approaches in a common large-scale benchmark. Its big amount of training samples as well as the challenging and representative test set enables the training of deep learning architectures and the extraction of reliable conclusions. The accuracy results (presented in Word Recognition Rate—WRR) from different methods are demonstrated on Table 4. It is worth noticing that the state-of-the-art performance has been raised by about 25% in only 5 years. Moreover, some general directions about an effective LR system can be deduced by analyzing the previous works presented on Table 4. In this context, the components of a LR system that have a significant effect on the performance can be determined, even though each method has its own customized settings for training and image processing. The first observation can be summarized around the importance of 3D convolutional layers since the usage of the spatiotemporal convolutions at the initial layers of the topology is dominant and boosts the performance. The second observation is related to the selection of the Deep CNN in the frontend, where ResNet architectures demonstrate to have a positive impact. The third observation can be made about the necessity of a sequence encoding topology at the backend, either with recurrent architectures, like (bi-)LSTM and (bi-)GRU, or recently with TCNs. Whilst considering the above three guidelines into the design of a LR system can result a classification accuracy of about 82–83%, further increasing of the performance is difficult and requires a different strategy in the frontend. Thus, an efficient approach is the 2-stream networks using both grayscale image and optical flow or deformation flow inputs [19,20,31,33]. Moreover, the 3D augmentations of the training images for generating synthetic facial data in arbitrary poses enable the system to generalize well and can further improve the performance [34]. The (local and global) mutual information maximization constraint enhances the ability of the CNN model to learn fine-grained lip movements and discover key frames [32]. Furthermore, the work of Courtney et al. [41] reveals the power of alternating blocks of convolutional and LSTM layers along with residual connections for increasing the performance. In addition, the influence of the preprocessing in the input image must be highlighted too. By introducing a normalization procedure, such as the face alignment (aligned mouth), the accuracy overcomes 85% since the models are able to learn in a more consistent manner [16,17,22]. Similarly, the pretraining on another LR dataset [41] or even on a different task [20] can lead to improved results. Driven by our prior findings [21], the proposed LR system extends the works based on TCNs considering the recent advances, which replacing the recurrent topologies with temporal convolutional networks in the backend can achieve similar or even better performance than LSTM or GRU with less computational cost [22]. Thus, we incorporate the simple version of MS-TCNs in the backend as well as utilize the 3D convolutions via ALSOS module together with the efficient ResNet-18 topology in the frontend. Our three ALSOS-based LR systems of Figure 3 are evaluated on the LRW-500 dataset, and the WRR results are presented on the Table 4 below.

A fair comparison between all methods can be a very difficult task due to many different technicalities, like the input image size, the data managing policy, or the exploitation of pretraining in another dataset and task, which impact the performance. However, it must be kept in mind that all methods are evaluated on the common and challenging test set of LRW dataset. It is obvious, regarding the Table 4, that the improvement between methods can be relatively small in terms of accuracy values; nonetheless, it is noteworthy because of the very large test set (i.e., 25 thousand samples) where even a small difference means a significant number of correct predictions. It is useful to note here that a look at the recent works that evaluated the LRW dataset indicate simply the classification accuracy as the only reported result [16,17,22,32,33] while the others use it as the predominant metric (with the proportion of times that the correct class is found in the top-K predictions for the word and not only when K = 1, top-1) [24]. Hence, our feel is that the proposed LR system achieves an accuracy 87%, which is at least comparable with the state-of-the-art results. Given the various technicalities of the different works, we eventually select to follow the experimental protocol method and implementation of Martinez et al. [22] in order to accomplish a fair direct comparison with this work leastwise. As a consequence, the superior result of our approach (i.e., 85.65%) against the method from [22] (i.e., 85.30%) indicates the effectiveness of the ALSOS module in the performance as long as only the spatiotemporal stage (3D) is replaced with the ALSOS module. In the next experiment, the frontend of the LR system is converted while the backend kept unchangeable, and the classification accuracy is increased up to the value of 87.01%. Thus, the importance of ALSOS can be summarized in the properties of efficiency, since all three variations achieved high performance of more than 82%, and practicability, since it is a self-contained module that can be used in a plug-and-play logic inside the framework. Moreover, the implementation of ALSOS leads to some interesting conclusions. First, the contribution of ALSOS module is mainly observed when used in different levels of the ResNet-18 architecture. This is compliant with our initial assumption that the ALSOS-based topology will gradually summarize spatial and spatiotemporal information. This is in line with the implementational structure of the LR system proposed by Courtney et al. [41] but without the complexity of alternating Bi-LSTM blocks. While further investigation and research experiments beyond Lip reading are required to shed more light in this finding, it is obvious that 3D convolutional layers generalize better when used in later stages of a CNN, something that can be explained from the fact that the weight of the first layers in a convolutional architecture is the most difficult to learn. This problem is detected also from Weng et al. [20], and they solved it by performing a pretraining on an auxiliary large video dataset, like Kinetics, which requires a vast amount of computational resources. However, we overcame the overfitting problem of ALSOS without the need for an external database, by alternating the ALSOS with ResNet blocks and taking advantage of the corresponding skip connections to solve the vanishing gradient problem. After all, the achieved high classification accuracy values from all the proposed ALSOS and ResNet variants confirm that the ALSOS module is advantageous for the Lip-reading task.

Additionally, we found important to mention the top-3, top-5, and top-10 accuracies, following the metrics proposed by the designers of the dataset [24]. These scores provide a clearer regarding of the ability for our three proposed LR systems. Under the top-K evaluation, a prediction is accepted if the first K predicted labels contain the correct class. The results are summarized in Table 5. The alternating ALSOS network achieved the best performance in all top-k evaluations, demonstrating the consistency and robustness of this approach.

## 5. Conclusions

In this work, we presented a LR system focusing on the design of a novel module based on spatiotemporal convolutions. The proposed module is named ALSOS, and it alternates between 3D and 2D convolutions in order to summarize the spatial and temporal information gradually, enabling 2D CNNs to operate independently on each image in between ALSOS modules. In this work, the ALSOS module is inserted in the ResNet architecture and therefore encapsules the spatial and temporal dependency either from the input image sequence or intermediate feature maps of the ResNet, allowing the system to hierarchically encode spatiotemporal information. The contribution of the proposed module was evaluated in depth, by investigating the position of ALSOS in any place of the LR topology, something that is feasible due to the portable self-contained capability of the ALSOS module. We showed that the 3D convolutions stage is crucial for the overall performance since all variations of ALSOS—attached in different positions—provide adequate results; however, the blend of ALSOS and ResNet blocks performs the best. Overall, the proposed system for word-level Lip reading composed of the 3D and 2D convolutional (ALSOS and ResNet) frontend along with 1D convolutional (TCN and FC) backend was evaluated successfully on two different languages. Thus, our LR system obtained high results with the popular English LRW-500 dataset as well as it coped successfully with real-world biomedical LR tasks using the Greek LRGW-10 dataset.

## Figures and Tables

**Table 1:** Number of operations and processing time for LR systems.

LR System	GMACS	Time (ms)	Parameters
ALSOS + MS-TCN	72.37	56.9	28.22M
ALSOS & ResNet-18 + MS-TCN	12.45	16.1	40.12M
3D & ResNet-18 + MS-TCN [22]	10.31	11.5	36.36M
Alternating ALSOS & ResNet-18 + MS-TCN	14.49	13.8	41.23M

**Table 2:** Classification Results on the LRGW-10 medical oriented dataset.

Method			Data	LRGW-10
Authors’ Name (Year)	Frontend	Backend	Input Image Size	Classification Accuracy (%)
Kastaniotis et al. (2020) [21]	3D & ResNet-18 (pretrained on ImageNet)	MS-TCN (He Normal initialization)	112 × 112	41.1
Kastaniotis et al. (2020) [21]	3D & ResNet-18 (pretrained on subset of LRW)	MS-TCN (pretrained on subset of LRW)	112 × 112	50.2
Ours	ALSOS (He Normal initialization)	MS-TCN (pretrained on LRW)	112 × 112	44.2
Ours	ALSOS & ResNet-18 (pretrained on LRW)	MS-TCN (pretrained on LRW)	112 × 112	51.6
Ours	Alternating ALSOS (He Normal initialization) & ResNet-18 layers (pretrained on ImageNet)	MS-TCN (He Normal initialization)	112 × 112	51.8
Ours	Alternating ALSOS & ResNet-18 layers (pretrained on subset of LRW)	MS-TCN (pretrained on subset of LRW)	112 × 112	54.3
Ours	Alternating ALSOS & ResNet-18 layers (pretrained on LRW)	MS-TCN (pretrained on LRW)	112 × 112	56.3

**Table 3:** Classification accuracy (%) on the validation set of LRW-500 dataset when various mini-batch sizes are utilized in the training of the LR system.

Classification Accuracy (%) on the Validation Set of LRW-500				
Mini-Batch Size	8	16	32	40
Alternating ALSOS & ResNet-18 blocks	85.96%	86.1%	86.6%	87.0%

**Table 4:** Summary of the state-of-the-art results on the LRW-500 dataset.

Method			Data		LRW-500
**Authors’ Name (Year)**	Frontend	Backend	Input Image Size	Input and Data Managing Policy	Classification Accuracy Top-1, WRR (%)
Chung et al. (2016) [24]	3D & VGG M	-	112 × 112	Mouth	61.10%
Chung et al. (2017) [42]	3D & VGG M version	LSTM & Attention	120 × 120	Mouth	76.20%
Petridis et al. (2018) [43]	3D & ResNet-34	Bi-GRU	96 × 96	Mouth	82.00%
Stafylakis et al. (2017) [18]	3D & ResNet-34	Bi-LSTM	112 × 112	Mouth	83.00%
Cheng et al. (2020) [34]	3D & ResNet-18	Bi-GRU	88 × 88	Mouth & 3D augmentations	83.20%
Wang et al. (2019) [19]	2-Stream ResNet-34 & DenseNet3D-52	Bi-LSTM	88 × 88	Mouth	83.34%
Courtney et al. (2020) [41]	alternating ResidualNet Bi-LSTM	alternating ResidualNet Bi-LSTM	48 × 48, 56 × 56, 64 × 64	Mouth	83.40%
Luo et al. (2020) [31]	3D & 2-Stream ResNet-18	Bi-GRU	88 × 88	Mouth and gradient policy	83.50%
Weng et al. (2019) [20]	deep 3D & 2-Stream ResNet-18	Bi-LSTM	112 × 112	Mouth & optical flow	84.07%
Xiao et al. (2020) [33]	3D & 2-Stream ResNet-18	Bi-GRU	88 × 88	Mouth & deformation flow	84.13%
Zhao et al. (2020) [32]	3D & ResNet-18	Bi-GRU	88 × 88	Mouth and mutual information	84.41%
Zhang et al. (2020) [16]	3D & ResNet-18	Bi-GRU	112 × 112	Mouth (Aligned)	85.02%
Feng et al. (2020) [17]	3D & SE ResNet-18	Bi-GRU	88 × 88	Mouth (Aligned) & augmentations	85.00%
Martinez et al. (2020) [22]	3D & ResNet-18	MS-TCN	88 × 88	Mouth (Aligned)	85.30%
Ours	ALSOS (4 stacked)	MS-TCN	88 × 88	Mouth (Aligned)	84.38%
Ours	ALSOS & ResNet-18	MS-TCN	88 × 88	Mouth (Aligned)	85.65%
Ours	Alternating ALSOS & ResNet-18 blocks	MS-TCN	88 × 88	Mouth (Aligned)	87.01%

**Table 5:** Classification top-3, top-5, and top-10 accuracies (%) on the LRW-500 dataset.

Classification Accuracy (%) on the LRW-500 (top-K)			
LR System	Top-3 Accuracy	Top-5 Accuracy	Top-10 Accuracy
ALSOS (4 stacked)	86.8%	97.3%	98.3%
ALSOS & ResNet-18	88.4%	97.8%	98.7%
Alternating ALSOS & ResNet-18 blocks	88.7%	98.0%	99.3%

## Data Availability

No new data were created or analyzed in this study. Data sharing is not applicable to this article.
